# iVici: Interrelational Visualization and Correlation Interface

**DOI:** 10.1186/gb-2005-6-13-r115

**Published:** 2005-12-30

**Authors:** Kirill Tarassov, Stephen W Michnick

**Affiliations:** 1 Département de Biochimie and Centre Robert-Cedergren, Bioinformatiques et Genomiques, Université de Montréal, CP 6128, Succursale Centre-ville, Montréal, Québec, H3C 3J7, Canada

## Abstract

We have developed an application, iVici, to analyze cellular networks represented as addressable symmetric or asymmetric two-dimensional matrices. iVici was designed to permit simultaneous visualization and correlation of multiple datasets, representing any relationship between a set of genes, mRNAs, or proteins. Visual overlay of datasets and addressable access to gene annotations permits comparison of networks of different types (for example protein-protein interactions and genetic networks) or investigation of the dynamic reorganization of a particular network.

## Abstract 2

iVici, a new tool for the simultaneous visualization and correlation of multiple datasets, allows the analysis and comparison of different types of networks.

## Rationale

Efforts to utilize the rapidly growing number of large-scale datasets to describe the organization of cellular biochemical and genetic networks require sophisticated tools for graphical representation and comparison of experimental results. At the level of data analysis and hypothesis testing, heat map representations have proved to be among the most useful, for example as a general method for visualizing mRNA expression profiles [1]. They have also been broadly applied to many large-scale analyses, ranging from interpretation of complex relationships such as synthetic genetic interactions to physical coupling of functional interactions among amino acids in protein structures [2]. On the other hand, biochemical or genetic networks can be inferred from large-scale data represented as graphs of nodes connected with edges. Coloring and grouping of nodes on circular layouts are used to integrate different data on the same graph. Software packages such as Osprey [3] and Cytoscape [4] can be used both to construct such networks and as interfaces to databases from which detailed biological inferences can be drawn.

Some limitations of these approaches call for complementary graphical representations of large-scale data. For instance, heat maps are limited to analysis of one dataset and type at a time, and graphs lose their intuitive value rapidly as nodes are added. In a number of recent reports networks of protein-protein interactions were represented as a two-dimensional matrix, in which rows and columns correspond to proteins and matrix values describe relationships between the proteins; clustering algorithms were then applied to identify network modules with similar interconnectivity [5,6]. The elegance and spatial properties of two-dimensional matrix representations suggest a number of ways in which these could permit rapid and intuitive visual analysis and interpretation of large-scale datasets with broad and general applicability. For instance, although two-dimensional matrix representations of hierarchically clustered data can be visualized with existing software such as TreeView, these do not permit comparative analysis of different datasets. Furthermore, these programs are not interactive, and do not allow scrutiny, for example, of genes or groups of genes with interesting relationships in comparative datasets. To fully take advantage of two-dimensional heat map representations and to facilitate the visualization and analysis of heterogeneous data, we have developed a tool - iVici (Interrelational Visualization and Correlation Interface).

iVici is a multiplatform program written in Java that is capable of simultaneous visualization of clustered matrices representing various biological datasets.

## Visualization modes

**Figure 1:**
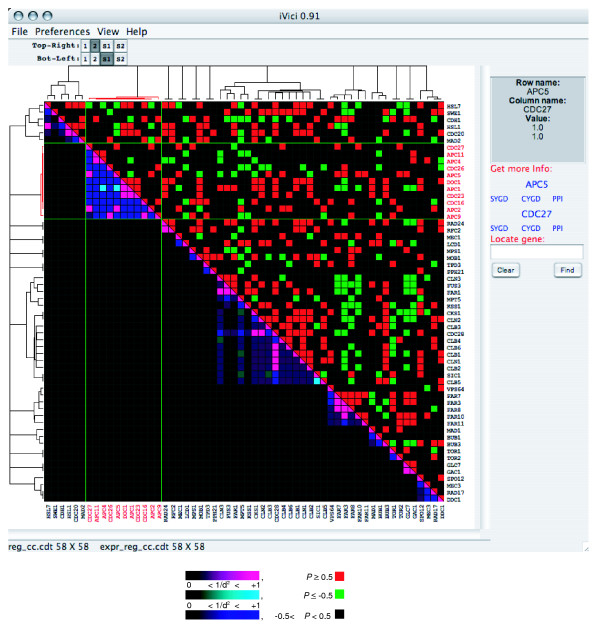
Representation of a network of protein-protein interactions and its correlation with mRNA expression profiles. The lower left triangle corresponds to a network of protein-protein interactions derived from small-scale experiments between 58 cell cycle regulators. For each two regulatory proteins, an association value was calculated as 1/d^2^, where d is the shortest path between the proteins in the interaction network [5]. The upper right triangle corresponds to correlation of mRNA expression profile changes during the cell cycle between pairs of cell cycle regulatory genes. Protein pairs that are colored red correspond to Pearson correlation coefficient (*P*) values greater than 0.5, and those colored green correspond to *P* values less than -0.5. In the bottom left triangle, an association value of two proteins that have a significant positive correlation coefficient in their mRNA expression levels is colored magenta (for positive correlation) or cyan (for negative correlation). Association values between uncorrelated proteins are colored blue.

iVici has three visualization modes: general, comparative and superimposed. The general mode is used to visualize hierarchically clustered results in the format generated by the software package Cluster [1]. In this mode, iVici is similar to traditional visualization software. Extended features of iVici allow one to link datasets to multiple web databases, to search and highlight specific row and column names, to add a grid to a heat map, and to customize effectively the graphical representation of results (Figure 1).

The comparative mode is designed for compact side-by-side representation of symmetric data matrices, which are used, for example, to analyze pair-wise protein-protein interactions. Asymmetric datasets can also be visualized and overlaid using the general and superimposed modes, but not the comparative mode. Because of identical clustering in two dimensions, symmetrical matrices contain the same information above and below the main matrix diagonal. Thus, two datasets can be represented as triangles and fitted into the same matrix square. iVici will use the first loaded dataset as a reference, and the second dataset will be organized according to the reference, where data value j, i (j > i) from the second dataset corresponds to the i, j value from the first dataset. Superimposed mode is designed to provide a visual representation of data intersection. iVici allows one to choose different colors for color scale rendering of data values from two datasets. In superimposed mode, when a non-zero value exists in both datasets, a superimposition of two dataset colors is used for color scale rendering. Non-zero values that are present in only one of the datasets are displayed in the original dataset color.

In Figure 1, a network of protein-protein interactions is compared with the correlation between mRNA expression profiles. The bottom left triangle corresponds to a network of pair-wise small-scale interactions taken from the CYGD [7] database between *Saccharomyces cerevisiae *proteins that are annotated as regulators of the cell cycle (RCC) according to Gene Ontology (GO) annotation [8] (RCC network). For each two regulatory proteins, an association value was calculated as 1/d^2^, where d is a shortest path between the proteins in the interaction network [5]. The highest association value is 1, which corresponds to direct interaction between two proteins and results in maximum color intensity on the heat map representation. In the upper right triangle, rows and columns correspond to the network proteins and protein pairs are colored corresponding to Pearson correlation coefficients (P) greater than 0.5 (red) or less than -0.5 (green) calculated for changes in mRNA levels during the cell cycle (see Spellman and coworkers [9] for synchronized cells selected by elutriation).

In order to facilitate comparison of two datasets, a superimposed color scheme is used for the lower left triangle. An association value of two proteins that have a significant correlation coefficient is rendered in magenta (for positive correlation) or cyan (for negative correlation). These colors are combinations of the primary blue used in the first dataset and the red and green colors used in the second dataset. The blue color is used to represent an association value of uncorrelated proteins. Such representation permits coloring of correlated elements while preserving the original structure of the network. For example, in Figure 1 subunits of the anaphase-promoting complex form a cluster, which is highlighted in green. Below the main matrix diagonal, protein-protein interactions are visualized. The iVici superimposed mode is used to highlight in magenta those interacting protein pairs that exhibit significant correlation in terms of changes in mRNA expression levels during the cell cycle (for example, pairs APC1-CDC23 and APC5-CDC27). These relationships are immediately apparent and unambiguous. Existing software packages for visualization of heat maps do not permit such coloring, and one must use other graphical software to prepare visual representations of correlated data, which is in itself a laborious process.

## Correlation of multiple data sources

iVici does not require pairs of datasets to be completely overlapped, thus allowing visualization of correlations between data that are incomplete or missing data points. Matrices of different dimensions and with an arbitrary order of rows and columns can be loaded into the application, which will automatically align the datasets with each other based on column and row names. In order to keep the visualization color scheme simple, comparative and superimposed modes are used to display two datasets at a time. However, when it is necessary to correlate more than two datasets, an export feature of iVici can be used to display the overlap between the first two datasets in a separate application window, where it can be further compared with other datasets.

**Figure 2:**
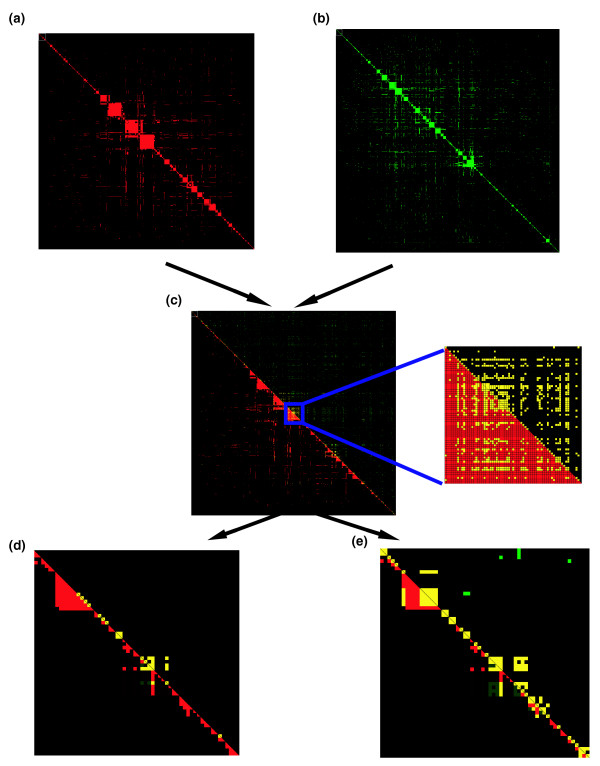
Correlation of multiple datasets. Two protein interaction networks derived from large-scale experiments conducted by (**a**) Matrix of 1,349 by 1,349 interacting proteins from Gavin and coworkers [10] and (**b**) Matrix of 1,550 by 1,550 interacting proteins from Ho and coworkers [11]. These interactions are compared with the protein interaction network of regulators of the cell cycle (RCC; in panels d and e). (**c**) A supperimposed matrix. The two large protein interaction networks are overlaid and interactions common to both networks are highlighted in yellow. Interactions that are found only in one of the networks are colored red (interactions found only in the data reported by Gavin and coworkers [10]) and green (interactions found only in the data reported by Ho and coworkers [11]). (**d**) An overlap of the two interaction networks is compared with the RCC network. Interactions of the RCC network that are found in both large-scale pull down studies are highlighted in yellow. (**e**) A union of the two protein interaction networks is correlated with the RCC network. Interactions of the RCC network that are found in at least one protein interaction network are highlighted in yellow. Red is used to color the RCC network interactions that are not found in either protein interaction network. Interactions present in one of the protein interaction networks but not found in the RCC network are rendered in green.

As an example of correlation of multiple data sources, in Figure 2 the network of protein interactions between 58 cell cycle regulators (RCC network), described above, is compared with two protein-protein interaction networks from large-scale pull down experiments [10,11]. In panels a and b of Figure 2, networks of protein interactions derived by Gavin and coworkers [10] and Ho and coworkers [11] are represented. In order to discriminate between these two datasets, interactions in Figure 2a are rendered in red and those in Figure 2b are rendered in green. We assigned an interaction between two proteins if they were found to be in the same complex in the corresponding study. In Figure 2c the overlap between the two networks is visualized in superimposed mode. Interactions confirmed by both large-scale pull down studies are colored in yellow. Interactions that are found only in the data reported by Gavin and coworkers [10] are colored red and those found only in the data reported by Ho and coworkers [11] are colored green.

In order to compare these protein interaction networks with the RCC network, it is possible to visualize either an overlap (Figure 2d) or a union (Figure 2e) of datasets represented in Figure 2c. An overlap of two large-scale networks contains only interactions that were found in both networks. In Figure 2d, interactions of the RCC network that were found in both large-scale pull down studies are colored in yellow. In Figure 2e the RCC network is compared with a union of the two interaction networks. Interactions colored in red represent RCC network interactions that are not present in either protein interaction network. Yellow is used to color interactions in the RCC network that were found in at least one protein interaction network, and green highlights the interactions that were present in one of the protein interaction networks but not in the RCC network.

## Implementations and advantages

iVici can be used to conduct comparative analysis of any type of biological information that can be represented in both symmetrical (Figure 1) and asymmetrical matrix form [12]. Combinations of colors to represent datasets and color scale rendering of matrix values allows quick identification of regions of data intersection and preserves quantitative information describing relationships between entities in the datasets.

Representation of networks in two-dimensional matrix form has a number of advantages over traditional graphs of nodes and edges. The topology of the matrix overcomes the problem of visually overlapping information, such as graph edges, on complex networks. Even for a large number of highly connected nodes, the information on a particular feature is easily accessible in the corresponding matrix rows and columns. Moreover, specific information can be encoded in different parts of the matrix. In Figure 1, hierarchical clustering, performed symmetrically in two dimensions, was used to order genes according to their patterns of interconnectivity. The modular structures along the main matrix diagonal describe relations within the group, whereas off-diagonal elements represent relationships between distinct groups of genes. As an alternative to hierarchical clustering, ordering can be done according to positions of genes on chromosomes, direction of information flow in signal transduction or genetic networks, and subcellular compartments, among other options.

Because of the absence of visual overlap discussed above, modular structure in networks can easily be visualized, even for complex datasets. Furthermore, one can infer the biological relevance of modular structure by comparing different datasets for the same genes. For example, proteins that form modules of physically interacting proteins are expected to be co-localized or to perform a common biological function. These expectations may serve for testing unsupervised or supervised methods for ordering matrix entities, such as hierarchical clustering or other inter-relational analysis strategies.

Finally, iVici provides a simple way to capture the dynamic evolution of a biological process. For instance, it is simple to follow changes in pair-wise gene expression and/or protein turnover, as well as in a specific cellular compartment, that occur as a result of an intrinsic process (for example, cell or metabolic cycles) or specific perturbations of the cell. A zero-point representation of gene-protein pair relationships could be represented on the upper diagonal half matrix and the relationship at another point (for example, in time) in the lower half matrix. Changes in the relationships would then be visualized as specific colors for those gene/protein pairs for which a change has occurred.

## iVici download

iVici software version 0.9 for Mac OSX, Linux, and Windows, for use in nonprofit organizations, can be downloaded from the iVici website [13].
